# Rhetoric Versus Reality – Embedding a New Relationship Within Integrated Care Systems for Third Sector Organisations

**DOI:** 10.5334/ijic.8989

**Published:** 2025-06-27

**Authors:** Christopher Phillips

**Affiliations:** 1 Somerset Council and NHS Somerset ICB, UK

**Keywords:** third sector, partnership working, integrated care

## Abstract

**Introduction:**

Within the UK, NHS England has outlined the integral role of third sector organisations as a strategic partner in integrated care systems. This study sought to explore the embedding of a ‘new relationship’ in the co-design and delivery of ‘local’ services.

**Methods:**

Thirteen semi-structured interviews were conducted within a local authority area in England, with leaders from both the statutory and third sector. Interviews were analysed using framework analysis.

**Findings and Discussion:**

Findings suggest there is a need to go beyond the rhetoric in embedding a ‘new relationship’ with the third sector. More needs to be done to change the narrative as to how the third sector is perceived, for sectoral stereotypes to be dispelled, to move beyond tokenistic engagement and focus on how improving health can be tackled together. Whilst place-based forms of governance will differ, a greater understanding by the statutory sector of ‘local’ organisational and individual dynamics, capabilities and perspectives is paramount.

**Conclusion:**

The study concludes that policy narratives are not underpinned with institutional structures and mechanisms. Without a concerted effort and commitment to meaningful engagement, there is a risk that third sector goodwill dissipates in the face of the latest iteration of policy rhetoric.

## Introduction

### International Context

Health and care systems globally are facing unprecedented pressures of increasing needs and demands from an ageing population, limited financial resources and an overburdened workforce [1], with health inequalities being further exacerbated by the COVID-19 pandemic. The World Health Organisation’s [2] global strategy on integrated people-centred health services calls for a ‘fundamental paradigm shift in the way health services are funded, managed and delivered.’ It argues if a shift in approach is not adopted then services will become increasingly ‘fragmented, inefficient and unsustainable,’ and will not meet people’s needs and expectations. Integration, is a widely used concept and in the context of this research paper, represents a ‘joining up of traditional silos of care across (horizontal) and within (vertical) systems, organisations, services and service providers’ [1, p.2].

Health systems globally are at different stages in rolling out system-wide models for integrated care. Steele Gray et al [3] in their study of international models tried to capture key components and insights into how innovative models could be scaled up, spread and sustained. Whilst recognising, as Shaw et al [4, p.20] outline ‘there is no one model of integrated care that is suited to all contexts, settings and circumstances,’ it is vital to consider contextual differences before implementing models in different services and locations [5]. In addition, Kee et al [6, p.2] draw attention to the impact of individuals on approaches, in referring to the ‘human dimension’ and so there is a need to look beyond structures and systems. In part owing to the ‘heterogeneity of local systems being evaluated’, Page et al [1, p.1] conclude that the ‘evidence base for integrated care in an English context is unclear.’

### Integrated Care Systems in England

In England, integration of care has been a long-standing policy concern. This is most recently set out in the NHS Long Term Plan [7] and the formal creation of 42 Integrated Care Systems (ICSs) as legal entities, as part of the Health and Care Act 2022. Each ICS being responsible for improving population health by taking a multifaceted and more collaborative approach, that tackles the ‘causes of the causes’, within a geographical area comprising of between 500,000 and 3 million people [8]. As part of this, NHS England [9] articulated the need to address the wider determinants of health through working with partners beyond health and care services. They, however, allowed the design and implementation of ICSs to be locally led within a broad national framework. Consequently, as The King’s Fund [10] sets out there are significant differences in the size and complexity of each ICS, and the maturity of partnership working across each will differ.

An expectation was placed on ICSs to develop arrangements for engaging and embedding voluntary sector organisations in decision-making arrangements and system-level governance, through the establishment of voluntary sector alliances which reflected the diversity of the sector. The voluntary sector or Third Sector Organisations (TSOs), as referred to in this study, were described as ‘a vital cornerstone’ by NHS England [11]. Arrangements within local ICSs were to be underpinned by a formal agreement which clearly sets out a ‘new relationship’ with the sector as:

A strategic partner in shaping, improving and delivering services, and developing and delivering plans to tackle the wider determinants of health [12, p.4].

The Department of Health and Social Care [13] similarly argued that to be effective, ICSs must be ‘a genuine partnership of equals’. Given Alcock [14] and Macmillan [15] both reference previous changes in the ‘political discourse’ regarding relations between the statutory and TSOs, the extent to whether this leads to a marked shift away from the existing ‘asymmetric’ power relationships is questionable [16]. Sinclair [17, pp.2–3] similarly questions the power dynamic in relationships and whether, despite the rhetoric, TSOs will continue to have a ‘mere presence, rather than a voice.’ He continues by questioning ‘how can organisations from different sectors and with different remits, perspectives and powers establish effective working relationships?’

Baird et al [18] argue whilst the third sector may be increasingly valued, its potential as an equal partner with the public sector has not yet been realised. To help explore this, Kara [19] has previously outlined the need to understand ‘local’ organisational dynamics and capabilities. More recently Mead et al [20, p.170] report there is limited research with regards to understanding ‘the complex environment in which national public health policy intentions are operationalised at a local level.’ For the purposes of this study, ‘local level’ is regarded as ‘place-based,’ synonymous with local authority boundaries, typically covering a population of around 250-500,000 [10].

NHS England [21] also acknowledge that capacity and infrastructure varies between and within geographical areas. National surveys [22,23] point to TSOs operating on a knife-edge, with concerns about financial sustainability, capacity and rising demands. Given health policies are ‘formed through the complex inter-relationship of context, process and actors’ [24, p.9], this study provided the timely opportunity to explore with a diverse range of senior stakeholders whether they were experiencing a ‘new relationship’ in the co-design and delivery of ‘local’ services. It enabled their insights on the challenges stakeholders face and their perceptions on partnership working to be articulated. In this context, the overarching research question for this study was:

What are senior stakeholder perspectives within a local authority area in England on a new relationship with the third sector in the emerging landscape of integrated care systems?

### Partnership working with the third sector and the role of individuals

There is considerable debate about what the voluntary or third sector is and how it should be defined. What is clear from the literature is that the ‘third sector’, or ‘voluntary’, or ‘not for profit’, or ‘volunteer sector’ is an umbrella term. It is distinct from both the public and the private sector, but it is also not one single homogenous group. It is as Chapman [25] outlines, made up of ‘independent, self-directed organisations which exist to serve purposes defined by themselves’, with organisations of different sizes working with different levels of complexity and they do not necessarily attend to issues in a ‘unified’ way. There has been increasing recognition, however, in policy debate and research about the importance of TSOs in supporting local communities to take a greater role in improving health and wellbeing. TSOs as outlined by Nelson et al [26, p.2]:

Can play an important role in developing and adapting services to their local context, representing and engaging underserved communities, addressing gaps in support…and involving volunteers in capacity building.

Following an international workshop in 2023, Nelson et al [26] reported the role and contribution of TSOs ‘as integrators of care’ is ‘insufficiently recognised and understood’ by global health systems and concluded that:

Meaningful engagement within integrated care is possible but requires attitudinal shifts, new working methods, rebalancing power within the relationships, and sufficient resources to support the collaboration [p.3].

Throughout the history of the third sector there is a constant tension between TSOs autonomy and accountability [16]. Paine et al [27], in their review of six comparative qualitative case studies in England, explore whether with the effects of the pandemic and the development of ICSs there is the potential to ‘reset’ relationships between the statutory and third sectors. They highlight the power imbalances experienced and identify five aspects shaping relationships, which they define as ‘policy, position, power, practice and people’. They acknowledge that policies can be translated in different ways as they are implemented, with ‘local’ practice differing in terms of how stakeholders may interpret rules and governance. Their study found considerable variation both within and between organisations noting that relationships are shaped by both the policy and practice in which they are situated, as well as by the resources which key actors have available. Paine et al [27] cite, for example, whilst the national policy declares the third sector as a ‘strategic partner’ their experience is that the emphasis still remains on TSOs being more a resource for service provision, with relationships dominated by commissioning processes and practices.

Perkins et al [28, p.46] found ‘partnerships require a deep commitment, space and time to develop, and a clear recognition of what they are trying to achieve while remaining attentive to changing circumstance.’ In their study they cite gaps in terms of trust, sound relationships and mechanisms to hold partners to account. Taylor-Robinson et al [29, p.6] similarly identify key barriers to partnership working including ‘cultural issues such as lack of shared values and language; and macro issues such as political and resource constraints.’ They cite the work of Hunter et al [30] which reported ‘partnerships being overwhelmed by the size of the agenda; difficulties of sustaining governance in the context of reorganisations; lack of trust; resource constraints; and tokenistic partnering.’

The King’s Fund [18, p.31] outline ‘successful co-production takes skill, time, confidence and mature relationships built on trust.’ Milbourne and Cushman [16, p.488–489] cite Bachmann (2001) in arguing ‘building and sustaining trust in inter-organisational relationships, may be challenging and more complex than often acknowledged in the literature.’ Milbourne and Cushman [16, p.489] further stress relationships between sectors are not ‘neat, boundaried or homogenous; they are multi-layered.’

White et al [31, p.195] similarly report the academic literature has predominantly focused on the strategic level, neglecting the role of individual practitioners and how ultimately, ‘collaborative working is actualised in relationships.’ This is supported by Van Meerkerk et al [32, p.3], who cite O’Leary et al (2012), in noting the role of the individual in collaborations receives limited attention. Body [33, p.256] adds commissioners are also often discussed in homogenous terms, ‘at times appearing to lack individual agency, voice and power.’

Given that health issues cross sectoral boundaries, Williams argues [34, p.105] for a need to strengthen inter-organisational capacity, while also recognising this is ‘unlikely to flourish in organisational structures that are based on hierarchal control and power.’ He refers to the importance of individual ‘boundary spanners’ who are working across traditional organisational boundaries. He notes they can be individuals from all levels, but they must be skilled in ‘relational and inter-personal attributes’ in order to build trust, sustain effective working relationships and operate in non-hierarchical environments. Kara [19, p.9] similarly cites ‘whilst partnerships are made up of organisations it is the individuals and individual dynamics which are key.’

## Methods

This article draws upon a qualitative study involving semi-structured interviews with senior stakeholders across both the statutory and third sector within a local authority area. Interviews took place in May and June 2022 and so ahead of ICSs being legally established on 1 July 2022. Given ICSs were in their infancy and the study was focused on assessing the implementation of polices from the perspectives of individuals whose organisations would be affected, applied policy research [35,36] was used to guide the study and questions.

Semi-structured interviews, informed by a topic guide were chosen, as they enabled ‘participant thoughts, feelings and beliefs’ to be explored in more detail [37]. The interviews were conducted online using Microsoft Teams and lasted between 45–60 minutes. The topic guide was informed by key themes emerging from the literature review and current policy documents. It was also shared with the countywide TSO infrastructure provider, drawing on their experiences of championing TSOs, to sense-check against other recent surveys of the sector and to help refine the overall focus and purpose of the study. The guide focused on contextual questions; individual’s perspectives and understanding of ICS developments locally; barriers and challenges in working across sectors; and relationships between the sector explored.

Interviewees were purposively selected by the researcher, with flexibility included for additional stakeholders to be identified through snowball sampling. Given the focus on system working, interviewees were leaders from across the local ICS and their lived experiences as professionals regarding partnership working, as opposed to individuals accessing services. Consideration was given to organisational type (local authority, health and third sector); seniority of role; and experience in partnership working between sectors (minimum twelve months). Owing to the size of the third sector, organisations operating within the local authority footprint with an infrastructural/support role were approached. Recognising, they would have greater oversight of the system and capacity to respond. Consideration was made to ensure this included different aspects of the sector (including physical activity, young/older people and mental health).

### Data collection

In total thirteen stakeholders were interviewed before confidence that ‘thematic saturation’ had been reached as no new thematic information was subsequently gathered [37]. Seven individuals currently worked in the statutory sector and six in the third sector, with five having experience of working in both, four were male and nine were female. The data collection and analysis were undertaken independently by the researcher, in order to comply with the academic requirements for the Master of Science degree in Public Health at the University West of England.

### Analysis

Reflexivity, through the use of a reflective log, was used throughout the study to allow for critical reflection and greater self-awareness [38]. Garry et al [39] encourage researchers to consider the ‘insider-outsider positionality debate’ when considering any potential impact on their research. In this instance, the researcher had an ‘insider’ position. Having worked within the local authority, they had both a ‘lived familiarity’ of the area and ‘a priori knowledge’ of some of the organisations involved in the study. This provided easier access to some of the research participants and an understanding of wider contextual issues. Although, the study still allowed for an external perspective given the researcher was not directly involved in the roll out of ICS proposals.

To ensure systematic and comprehensive data treatment, ‘framework analysis’ was adopted, given its ability to compare data both within individual cases and across cases [38]. Auto-transcription was corrected immediately following the interview and anonymised, with time stamps and line references added to support future cross-checking. Transcripts were ‘naturalised’ to reflect ‘intelligent verbatim’ [40] and notes were added to capture other ‘nonverbal cues’ [41]. A coding framework was developed, utilising both deductive and inductive codes and themes. This was based on the interview topic guide, literature review and subsequent interviews. Through NVivo 10 software line-by-line open coding was then adopted to inform a working analytical framework. The framework was refined and updated iteratively through the use of mind maps and further searching of the literature to compare and identify codes, themes and groupings used by other researchers [41]. It was then applied systematically to each transcript. An Excel spreadsheet was used to manage and summarize the data into a matrix comprising of one row per participant and one column per code. This enabled patterns both across and within cases to be clearly seen and any potential duplication to be identified.

### Ethics

Ethical consent was provided by the University of the West of England in May 2022 and approval to undertake the study was provided by the local authority’s Director of Public Health. All participants received an information sheet about the study prior to interview and informed consent was obtained, with no participants subsequently withdrawing their consent.

## Findings

The main themes from the interviews are outlined in Table One, then expanded upon in this section.

**Table One:** Main Themes.


NAME	REFERENCES

01. A ‘plethora’ of different alliances, groupings and interests	88

02. More of the same	126

03. Power imbalances	242

04. The role of individuals in fostering relationships	121

05. Barriers and challenges	98

06. Not like the glossy brochure	34


### A ‘plethora’ of different alliances, groupings and interests

The diversity of TSOs was a common theme throughout discussions, with concomitant challenges of the statutory sector not knowing who or how to engage with TSOs. This was recognised by some third sector interviewees (I-TSO) who referred to the third sector being like ‘a spectrum’ (I-TSO) and another (G-TSO) commented the statutory sector only have a ‘kaleidoscope view of everything,’ which is understandable given ‘we’ve got such a plethora of small organisations in our third sector’ (H-Stat). These insights are helpful in articulating the distinctiveness and diversity of the third sector and the importance that all ICS systems, both locally and internationally, consider and understand the heterogeneity of the sector. Thus, also recognising that the nature, size and capacity of TSOs will vary by geographical area. This is reinforced by interview B-TSO, ‘people don’t understand the breadth or what it is like to work in the third sector.’

Equally, it was noted that some of the third sector do not understand the pressures and challenges faced by the statutory sector and that they ‘just don’t have enough money to do all the things that they are mandated to do, let alone what they want to do’ (E-Stat/TSO). Although interestingly and whilst a small sample, given five of the interviewees had worked in both the statutory and third sector there is the implication that there may be broader understanding than others may realise. This is reflected in ‘because I come from the statutory sector, I actually know what it is like to have the bureaucracy’ (L-TSO/Stat). Therefore, reinforcing the importance of caution when it comes to using generalisations.

For there to be a ‘new relationship’, interviewees raised the need to address misconceptions, including TSOs do employ professionals, they are not necessarily cheaper and they are ‘not free’ (H-Stat). Given the broad spectrum of TSOs the importance of infrastructure and umbrella organisations was highlighted in supporting broader system join-up and acting as a ‘neutral broker’ to enable conversations with the third sector (A-Stat/TSO).

### More of the same

There was a sense across the interviews that the changes have not gone as far as they need to go and an underlying element of scepticism regarding a ‘new relationship’, captured by (A-Stat/TSO):

I sense a lack of ambition, a lack of clarity about what ICS locally is going to do differently and I am concerned that the ICS locally will be old wine in new bottles.

Meaning, ICSs may set out to change their organisational structures or present initiatives as new and innovative, but in reality will largely continue to deliver the same sorts of activities. The changes were seen as more ‘evolutionary rather than revolutionary’ (H-Stat), and that it is ‘still very much a health led system with the integrated part not yet fully defined’ (E-Stat/TSO). Interviewees noted a disconnection between rhetoric and reality, ‘too often it’s about a strategy document rather than something that works on the ground and actually delivers for people’ (A-Stat/TSO). An explanation offered for explaining the disconnection was that ‘the narrative might be there, but quite often the structures and mechanisms that are put in place don’t support what the narrative is’ (J-Stat). Many were keen to not revert back to previous working practices, but to build on the lessons from the COVID-19 response which they felt had helped ‘accelerate’ (I-TSO) relationships and demonstrated how the sectors could work differently together.

### Power imbalances

The dominant view from the interviews was that in viewing a ‘new relationship’, the suggestion that they are or will be ‘equal partners’ is misleading and ‘it still feels like a them and us and top-down approach’ (D-Stat). Others citing if it is to be equal then there should have been a conversation at the start about resourcing the sector involvement. There was a recognition that more should be done to invest in helping the third sector develop and investing in TSOs’ capacity and capability, particularly given the fragility of the sector and their dependency on short term funding arrangements is not conducive to strong and long-term relationships.

TSO interviewees raised that they go to a lot of meetings which they are not funded for and they have had to push for their voices to be heard. It was also recognised that sometimes there is ‘third sector representation for representation’s sake’ (B-TSO) and any new relationship should not be a ‘box ticking exercise’ (G-TSO). Others commented there is an over-reliance on ‘one or two individuals’ (F-Stat) with TSO representatives often significantly outnumbered on boards by statutory voices and so it is difficult to ‘advocate’ on behalf of the whole sector.

Another interviewee (E-Stat/TSO) framed equal partners as ‘a really good principle to aim for, but in practice it’s really difficult’, particularly if ‘one of us is paying the other to be engaged.’ Interestingly one interviewee (I-TSO) noted whilst ‘the temptation is to see the power flowing with the money,’ the third sector do hold power. This was recognised in terms of the insight TSOs bring, alongside the lobbying role they fulfil, which means TSOs can more easily than statutory partners speak out against national policy. Austerity has impacted on the amount of funding the statutory sector has available, but TSOs can also lever in funding from other sources which compliment statutory service delivery. Therefore, reinforcing the importance of playing to each other’s strengths and highlighting the added value the third sector can provide to any future relationship, as opposed to being ‘seen as some nice stuff within the community’ (B-TSO).

An underlying message across the interviews was a need to have clear system priorities, as currently activities felt disjointed and too much duplication. The impact of the political cycle both locally and nationally was recognised as making it difficult to have a sustained direction of travel. With concern raised that sometimes ‘things are changed for the sake of it, not because it will be better’ (A-Stat/TSO).

### The role of individuals in fostering relationships

In supporting partnership working the vital role of individuals and human connections was highlighted. Noting the risk ‘if one person in that relationship moves jobs then we are almost back to square one’ (A-Stat/TSO). Following recent statutory sector staffing changes this risk had materialised with one interviewee stating ‘we don’t really know who they are and there’s not a lot of connectivity’ (G-TSO).

The importance of being able to build relationships as a core soft skill was highlighted. Concern was expressed that with remote working, there is a risk this vital skill could be lost, as being able to meet face-to-face and ‘pick up the phone and have an open and honest conversation’ (F-Stat) is conducive to developing effective working relationships and overcoming ‘cultural barriers’ (B-TSO). Consensus across the interviews, was in order for any ‘new relationship’ to flourish there is the need for more time to be invested in getting to know each other better. Recognising ‘we each bring something different to the table, and the benefits of ‘bringing different experiences, knowledge and thinking’ (I-TSO). There was a feeling that ‘genuine third sector involvement is not yet structural, it’s aesthetic’ (G-TSO) and not part of everyone’s ‘mindset’ (K-Stat/TSO). The need to work together was summed up eloquently by one interviewee (L-TSO/STAT) ‘we’re all in it together, so let’s be in it together.’

### Barriers and challenges

Traditional commissioning and procurement processes were cited as a key barrier to any future relationship. They ’pitch organisations against each other’ (C-Stat), as opposed to enabling coproduction and collaboration, which is presented as at the heart of the policy shift to ICSs. The dominant view was that statutory services ‘have got bogged down in the bureaucracy, the structures, the processes and procedures.’ (K-Stat/TSO). Whilst it was acknowledged accountability for the ‘public purse’ is important, the need for streamlined decision making was highlighted (A-Stat/TSO). A consistent message was that TSOs are still very much seen as providers not partners.

The other big frustrations expressed by both sectors was system bureaucracies and not having enough time, capacity or financial resources to engage fully with each other. This inadvertently impacted on TSOs’ capacity to participate in meetings or commissioning activity. Similarly, for commissioners it impacted their ability to establish greater awareness and relationships with the third sector. Frustration was also expressed, due to the lack of system join-up, about wasting capacity. Examples were given of having to have ‘the same conversations three times’ (B-TSO) which places further strain on already limited capacity. This being further exacerbated by the fact the day job of a TSO leader is ‘a million miles away from anybody else’s round the table… I don’t have people to do stuff for me.’

### Not like the glossy brochure

When considering models of good practice, the importance of ICS systems understanding local context was clearly articulated. There was very little congruence across the interviews in terms of making use of good models or best practice from elsewhere. There was a scepticism amongst some that ‘it’s never quite as good as the glossy brochures make it out to be’ (K-Stat/TSO) and that ‘they can give you ideas and they can inspire you but the trick is in the application, the people and how they work together’ (A-Stat/TSO).

Positively, a local example was given where commissioners are reported as working more ‘collaboratively’ (D-Stat) with the third sector in commissioning mental health services. One interviewee noted ‘there’s no hierarchy in it’ (C-Stat) and another reflected on the importance of ‘bringing people together’ (E-Stat/TSO). This provides the possibility of learning from local examples which are perceived to be working differently. The need for models to evolve and respond to ‘local’ context, is evident in ‘what we’ve ended up doing isn’t necessarily what we thought we would be doing, but it works’ (I-TSO).

## Discussion

This study has highlighted the complex environment in which national health and care policy intentions, such as the roll out of integrated care systems, are operationalised at a ‘local’ level. It adds to the existing literature and whilst the study is focused on England, the findings are relevant for other countries developing system-based approaches to integration, by outlining the importance of changing the narrative; moving beyond tokenistic engagement; working across organisational boundaries and recognising the collective role of individuals in enacting change.

### Changing the narrative

This research supports the recommendation by Kara [19] of the need to move beyond ‘sectoral stereotypes’ and the importance of considering a diversity of perspectives. Recognising the third sector as a spectrum more positively reflects the diversity, uniqueness and added value of TSOs, but also helps explain the challenges all ICSs face in terms of knowing how and who to engage with, given the breadth of the sector.

It is an uneven landscape, with some TSOs having greater capacity or resources to engage, and so relationships within ICSs will vary [15]. Years of austerity has seen globally a reduction in third and statutory sector capacity. In order to support both financial sustainability and sector engagement, commissioners and third sector interviewees highlighted the need to invest in the strategic capacity and capability of the sector. One practical example is provided in a case study of Lancashire and South Cumbria’s ICS [42], which highlights the need for a participation budget to support TSOs engagement in meetings. Perkins et al [28, p.42] also outline, the need to establish greater ‘vertical and horizontal linkages’ between partners. In support of this, Wheeler et al [42] share the benefits the funding of an independent chair can bring in building and developing relationships. Often complementing and enhancing the role of third sector infrastructure organisations in helping to facilitate a more coherent system-wide approach. Carpenter et al [43], in their study of Oxfordshire’s ICS, add the benefits of nationally coordinated leadership programmes, where funding and facilitation support were made available to support co-production and place-based alliances.

### Engagement beyond tokenism

The interview feedback has provided further examples of what Jackson [44, p.17] referred to as a ‘mismatch’ between rhetoric and reality. Power imbalances continue to be present, with key TSOs feeling under-valued, which adds to the research by Milbourne and Cushman [16, p.489] who noted, ‘many organisational relationships encompass goodwill, but far fewer involve participants on an equal basis.’

ICSs are intended to be a fundamental departure from previous health structures with an enhanced role for the TSOs in decisions about resource use and prioritisation. However, as evident in the phrase ‘old wine in new bottles’ there is some scepticism about whether this is yet another public service restructure. Baxter et al [5, p.9] in their systematic review of international evidence similarly challenged whether integrated care initiatives lead to ‘unequivocally positive effects.’ Statutory guidance and a national commitment to working with TSOs are therefore not enough, particularly given partnership working has been central to public policy since the late 1990s [28]. System governance is ‘as much about behaviour, values and attitudes as about structures, systems, processes and controls’ [45, p.17]. Despite all commissioners recognising the value which TSOs provide, there are missed opportunities for join-up at a ‘local’ level [46], with TSOs expressing frustration at not being involved in conversations earlier enough and decisions often being ‘delivered’ to them (G-TSO).

Kara [19] warns against the assumption that the third sector can be represented by one or two members and cites Macmillan (2003) who makes a subtle distinction between ‘being a representative of the sector’ to being a representative ‘from’ and ‘for the sector.’ TSO interviewees also felt they needed to be assertive in meetings with the statutory sector in order to ensure their voice is considered thus reflecting the power dynamic in working relationships is not even.

A sense of a lack of ‘systems leadership,’ as framed by Bigland et al [47], was conveyed. A number of interviewees advocated for clearer priorities, which takes account of different perspectives, in considering collectively how health inequalities and improving population health can be addressed. To support this, both the existing literature [18,48] and the interviews outlined the need to engender a shared mutual understanding of each other’s roles. A key consideration for all ICSs and global systems is without investing time in understanding each other’s priorities and challenges there is a risk that partnerships will be ineffective and tokenistic [19].

### Working the boundaries

Interviewees expressed concern about slipping back into traditional working practices post pandemic and not being involved in supporting service design and delivery. Consideration is, therefore, needed as to how meaningful engagement between the different sectors can be institutionalised and sustained. The COVID-19 response demonstrated globally how TSOs could quickly adapt, digitalise their offer and enhance state capacity. Levine et al [49, p.17], in their global study, cite the vital role TSOs played in ‘filling the gaps’. Notably in improving food security, supporting medical care, distributing funds and supporting the flow of reliable information to communities. It saw the mobilisation of more informal grassroots action and highlighted how closer connections between the health sector and ‘hyper-local’ volunteer activity can help support vulnerable members within our communities, build trust and maximise the use of resources [43].

Working relationships are complex, with interviewees reporting positive relationships with staff of certain levels, but that it was not consistent across organisations. The importance of individuals and personalities was clearly evident with multiple interviewees advocating a need to address the soft-core skills of commissioners to support more collaborative practices. This aligns with the notion of ‘boundary spanners’ championed by Williams [34, p.115], who argues ‘the value of basic and effective oral, written and presentational communication skills cannot be overestimated.’ Although to be effective, individuals need to have the capacity in their roles to support integration and they rely heavily on local knowledge and contacts [50]. Bigland et al [47, p.3] outline the necessity of a collective and ‘concerted effort of many people working together at different places in the system and at different levels.’ There is a need in their view to ‘focus not just on the characteristics of individual system leaders, but also the organisational contexts in which they operate’, in order for ‘new relationships’ to be sustained. To succeed as a boundary spanner, Baaken et al, [51, p.7] highlight key interpersonal attributes, notably ‘empathy’, ‘sensitivity to power differentials’ and ‘persistence’. In addition, the need for individuals to be skilled in negotiation and complex problem-solving. From the interviews, the importance of an open mindset, honesty, actively listening and an underlying commitment to making connections were clearly apparent. Continual changes in points of contacts, within statutory organisations, therefore poses a risk to fostering more effective collaboration.

Frustrations expressed in the interviews about traditional commissioner and provider splits not being conducive to partnership working is a long-standing issue and risks undermining any future relationship. There is a need to move away from the principles of markets and competition and to challenge the ‘disproportionate power’ which ‘procurement’ hold in order to facilitate more collaborative approaches [52].

The King’s Fund [53] have helpfully identified key actions for all ICSs to tackle barriers and challenges impacting on partnership working, which can be applied globally and include testing different approaches to commissioning; sharing data and insight; providing funding and sustainable investment; supporting the development and evolution of third sector alliances. Further to recommendations for good practice [48], NHS England [12] have provided a simple checklist to support the embedding of TSOs in ICSs governance and partnership arrangements which could be utilised for benchmarking current arrangements and identifying priorities for action. There, however, remains a need to start somewhere. Bigland et al [47] refer to the underlying importance of relationships and progressing opportunities which present, by forming a ‘coalition of the willing,’ through bringing together key individuals from across the system.

### Strengths and Limitations

Whilst the focus on one geographical area may limit the generalisability of findings, this research provides new insights into a range of stakeholder perspectives on the ‘new relationship’ with the third sector in the emerging landscape of ICSs. With the proliferation of Non-Governmental Organisations (NGOs) and moves to bring them into local level systems of governance [54] this study clearly has implications for countries beyond the United Kingdom. Many global health systems are experiencing similar challenges in areas of power imbalances, lip-service to co-design and horizontal integration. It highlights the importance of all global health care systems considering the ‘local’ context, the importance of relationships, partnership dynamics and changing the narrative as to how the third sector is perceived. ICS formation was in its infancy at the time of this study. Given relationships and partnership working takes time to develop, there is a need for subsequent research and evaluation to track developments at future intervals.

A key challenge in compiling the research was the heterogeneity of the literature, in terms of its broad disciplinary basis. A mixed methods approach, as advocated by Conger [55], would have allowed for triangulation and added further rigour. Having a priori knowledge of some stakeholders arguably enabled additional insight to be sought and greater access to participants given professional relationships had already been established. To mitigate the risk of researcher bias, consideration was given to research positionality and the use of reflexivity, which enabled the researcher to reflect, analyse and challenge their own presumptions regarding the subject area. The use of framework analysis provided a systematic approach and transparency as to how findings were derived.

## Conclusion

International policy has framed the third sector as an integral part of local health care systems and as strategic partners. However, the findings from this research support the view that asymmetric relationships continue to prevail. The notion of ‘a genuine partnership of equals’, whereby TSOs feel valued or included and the collective resources of all partners are considered and utilised to best effect is yet to be actualised. More needs to be done therefore, if there is to be a ‘new relationship’ across all global ICS systems.

Any new relationship entails opening the space for TSOs to shape the fundamental priorities and processes, with a move away from framing TSOs as a resource for public sector commissioners to direct and control. There needs to be an emphasis on changing the narrative as to how TSOs are perceived, and for sectoral stereotypes to be dispelled. Alongside, a greater focus on how improving health and tackling health inequalities can be tackled together. The third sector consists of a myriad of organisations with diverse, and sometimes competing interests. Therefore integrated systems of governance need to avoid homogenising or suppressing diversity of voices. There is frustration, with TSO representatives feeling under-valued and their strengths not fully utilised. This is not limited to just TSOs, with a number of commissioners also expressing frustration with resource and organisational constraints they are faced with.

Those wishing to study the impact of ICSs would benefit from conducting comparative studies across different geographical and international landscapes to better understand the influence of ‘local’ organisational dynamics, capabilities and perspectives; and explore the extent to which policy narratives are being underpinned with institutional structures and mechanisms over time. There is a need to further explore the role of individual relationships in enabling organisations to work more effectively both across and within systems. With consideration also given to understanding the underlying traits, knowledge and skills required.

The COVID-19 pandemic shone a spotlight on the third sector. It saw a marked shift in TSOs being involved in collectively finding ‘local’ solutions. There is a need, therefore, to harness the positives of collective action and maximise the insights and strengths of all system partners, if a ‘new relationship’ is to be realised and sustained. TSOs globally are operating within the context of unprecedented change and competing demands for their time and capacity, with concerns for their financial sustainability. Without a concerted effort and commitment to meaningful engagement there is a risk that TSOs’ goodwill dissipates in the face of the latest iteration of policy rhetoric.
